# NMR studies of excluded volume interactions in peptide dendrimers

**DOI:** 10.1038/s41598-018-27063-3

**Published:** 2018-06-11

**Authors:** Nadezhda N. Sheveleva, Denis A. Markelov, Mikhail A. Vovk, Maria E. Mikhailova, Irina I. Tarasenko, Igor M. Neelov, Erkki Lähderanta

**Affiliations:** 1 St. Petersburg State University, 7/9 Universitetskaya nab., St. Petersburg, 199034 Russia; 2 Institute of Macromolecular Compounds, Russian Academy of Sciences, Bolshoi Prospect 31, V.O., St. Petersburg, 199004 Russia; 3 St. Petersburg National Research University of Information Technologies, Mechanics and Optics (ITMO University), Kronverkskiy pr. 49, St. Petersburg, 197101 Russia; 4 Laboratory of Physics, Lappeenranta University of Technology, Box 20, 53851 Lappeenranta, Finland

## Abstract

Peptide dendrimers are good candidates for diverse biomedical applications due to their biocompatibility and low toxicity. The local orientational mobility of groups with different radial localization inside dendrimers is important characteristic for drug and gene delivery, synthesis of nanoparticles, and other specific purposes. In this paper we focus on the validation of two theoretical assumptions for dendrimers: (i) independence of NMR relaxations on excluded volume effects and (ii) similarity of mobilities of side and terminal segments of dendrimers. For this purpose we study ^1^H NMR spin-lattice relaxation time, *T*_1H_, of two similar peptide dendrimers of the second generation, with and without side fragments in their inner segments. Temperature dependences of 1/*T*_1H_ in the temperature range from 283 to 343 K were measured for inner and terminal groups of the dendrimers dissolved in deuterated water. We have shown that the 1/*T*_1H_ temperature dependences of inner groups for both dendrimers (with and without side fragments) practically coincide despite different densities of atoms inside these dendrimers. This result confirms the first theoretical assumption. The second assumption is confirmed by the 1/*T*_1H_ temperature dependences of terminal groups which are similar for both dendrimers.

## Introduction

Dendrimers are highly branched monodisperse macromolecules with a well-defined structure. The main difference between dendrimers and other nanoscale molecules is the possibility to control their chemical composition, size, and architecture. It allows designing structures with unique properties for various applications.

In recent years poly-L-lysine (PLL) dendrimers have attracted attention from researchers due to their high biocompatibility and relatively low toxicity in comparison with most of other dendrimers^1^. They were used in many biomedical application, in particular as carriers for drug and gene delivery^2–6^.

The structural properties of lysine dendrimers were studied both experimentally^7,8^ and by computer simulation using molecular dynamics^9–12^ and Brownian dynamics methods^13^. In our papers^10,11^ we applied algorithms and computer programs elaborated by us for charged and branched polymers earlier^14–17^.

More general peptide dendrimers could contain not only lysine but also other amino acid residues. Such branched peptides were synthesized^18–20^ and used for several biomedical applications including drug and gene delivery, artificial enzymes, antimicrobial peptides etc^21–26^. In the last years the properties of the group of peptide dendrimers of the same topology but of different amino acid composition have been compared for possible use in DNA and siRNA delivery^27,28^. In the review^29^ the use of peptides for drug and gene delivery was also discussed. Beside experimental works on application of peptide dendrimers, there are several papers where computer simulation of peptide dendrimers was performed using molecular dynamics method^30–32^. In our opinion, peptide dendrimers have almost unlimited potential for use in biomedicine, since they allow the creation of a huge number of artificial branched peptides, the number of which is even greater than the number of natural (linear) peptides and proteins.

NMR relaxation methods are widely used to study the local mobility in macromolecules (see review^33^). Over the last 15 years, the description of NMR relaxation in dendrimers in dilute solution has been developed in theoretical^34^, computer simulations^35–38^, and experimental^39–43^ works. These results were summarized in the recent review by Markelov et, *et al*.^44^. It was established that the local orientational mobility of NMR active groups in a dendrimer depends on their topological distance from the periphery. This regularity is practically independent of the excluded volume (EV)^38^ and the hydrodynamic interactions^45^. Note that in ref.^38^ the EV effects for dendrimer segments were simulated by using the Lennard-Jones potential. Moreover, computer simulations demonstrated that EV effects only rotation of a dendrimer as a whole and this influence depends on the dendrimer size^38^. The rotation of a dendrimer as a whole was calculated from the finite slope of orientational autocorrelation functions for vectors from the dendrimer core to a terminal segment. Effect of EV is small for terminal groups and is big for groups in the dendrimer core^37^. However, the semiflexibility of dendrimers results in suppression of EV effects on the orientational mobility in the dendrimer^38^. We would also like to note recent studies of orientation mobility in dendrimer melts^46–49^.

The theory of orientational mobility has been developed for classical (“ideal”) dendrimers where there are no defects in the tree-like structure. The next logical step is the development of the theory of orientational mobility for non-regular hyperbranched polymers in which regular tree-like structure is not expected. Branching points in this kind of polymers could have different functionality and branches containing a random number of generations^50–53^.

In the first theoretical papers on branched macromolecules with side fragments, the viscoelastic models without EV were used^54–58^. It was obtained that for this model, the local mobility of the side fragments and mobility of the terminal segments are equal. This statement follows from the conclusion that the mobility of a dendrimer segment is determined by its topological distance from the periphery. In the terminology used, the side fragment is a terminal one. In real systems, such behavior would be observed if suppression of excluded volume interactions occurs, which the developed theory does not take into account.

Therefore, the aim of this work is to experimentally verify the following theoretical conclusions:

1. Excluded volume effects do not influence the orientational mobility of groups observed by NMR relaxation;
2. The mobility of groups in the side fragments coincides with the mobility of the terminal groups.

**Figure 1:**
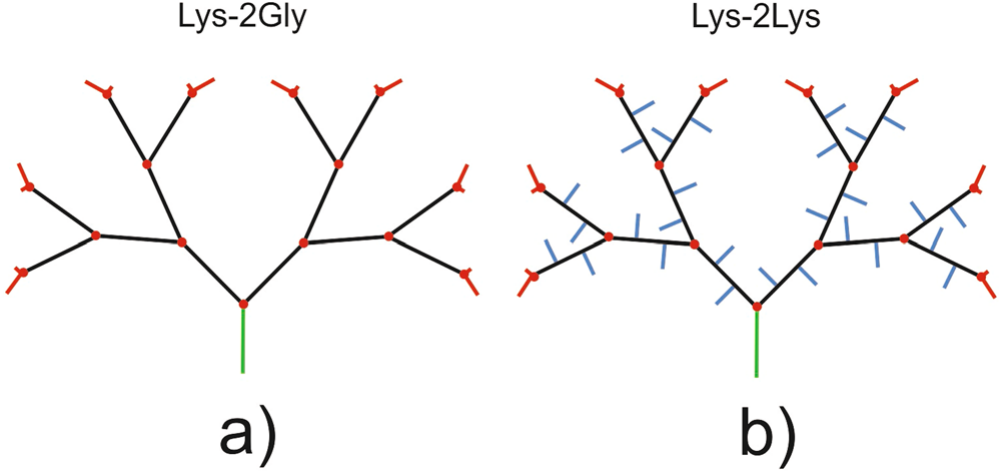
Schematic structures of the second generation (**a**) Lys-2Gly and (**b**) Lys-2Lys dendrimers. The colors mark following dendrimer parts: cores (green), inner segments of main chains (black), side fragments (blue), terminal segments (red). Red solid circles are the branching points.

**Figure 2:**
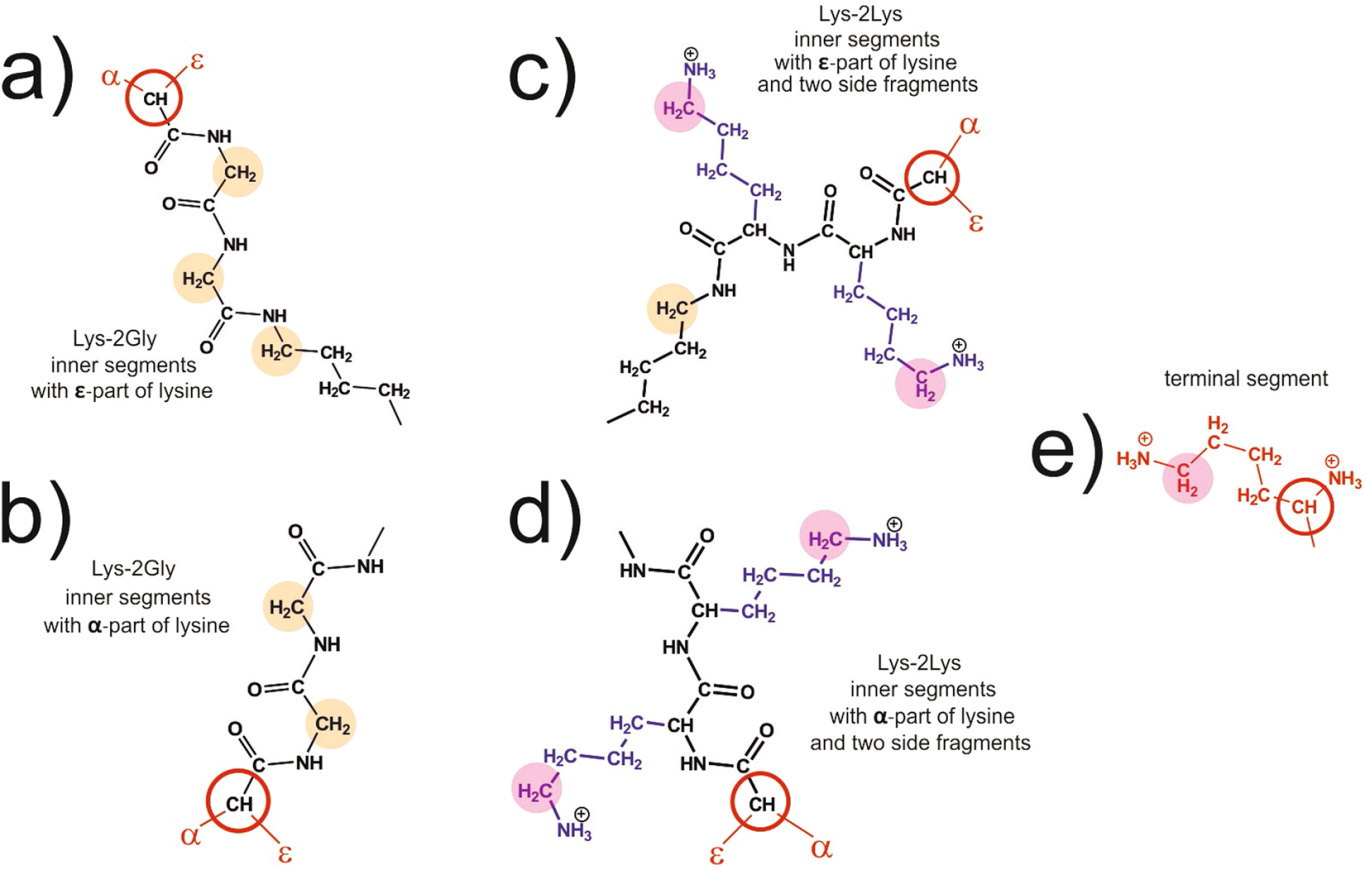
Chemical structures of inner and terminal segments in the Lys-2Gly and Lys-2Lys dendrimers. Lys-2Gly inner segments containing (**a**) ε-part and (**b**) α-part of lysine and two glycine residues; Lys-2Lys inner segments containing (**c**) ε-part and (**d**) α-part of lysine and two lysine residues; and (**e**) the terminal segment is the same for both dendrimers. Black color marks the main chain, blue one corresponds to the side fragments. Red open circles show the branching points. NMR active methylene groups of the main chain connected with NH groups are highlighted in orange color and CH_2_ groups connected with protonated NH_3_^+^ groups are highlighted in magenta color.

In this paper, we study two types of second generation peptide dendrimers (*G* = 2, 16 terminal groups) that are suitable for these purposes. The first dendrimer consists of repeating units containing one lysine residue and two linear glycine residues between each two neighboring branching points (Lys-2Gly-dendrimer) (Fig. 1a). The second dendrimer consists of repeating units containing three lysine amino acid residues one of which is branched while two other lysines are linear and connect neighboring branching points (Lys-2Lys-dendrimer) (Fig. 1b). Each segment of the Lys-2Lys-dendrimer comprises two side fragments containing the ε-part of lysine (Fig. 2c,d).

Both dendrimers have an asymmetrical branching (see Fig. 2). The contour lengths of the segments between the branching points in both are equal.

According to Fig. 2, Lys-2Gly-dendrimer has the CH_2_-(NH) groups inside the main chain and we consider them as “inner” groups in this study. Also, the Lys-2Gly-dendrimer has the “terminal” CH_2_ groups of lysine residue connected with terminal protonated NH_3_^+^ groups, which are located in terminal segments. Lys-2Lys-dendrimer has the “inner” CH_2_ groups in the main chain, but only in those segments, which contain the ε-part of lysine. In Lys-2Lys-dendrimer both CH_2_ groups connected with protonated NH_3_^+^ groups in the side fragments and in the terminal segments are considered as “terminal” one.

Due to an absence of charged NH_3_^+^ groups in the inner segments, the interior of the Lys-2Gly-dendrimer is less hydrophilic compared to the interior of the Lys-2Lys-dendrimer. We assume that it should lead to a more compact structure of the Lys-2Gly-dendrimer and, consequently, to the intensification of EV effects. Comparison of NMR relaxations of groups in the main chain of inner segments of Lys-2Gly and Lys-2Lys allows us to estimate the effect of excluded volume interactions and to check the first statement.

For the second statement, the same structures distinguished by the presence of side fragments help to evaluate the mobility of the segments.

We also present data for usual G2 lysine dendrimer, containing only one branched repeating lysine residue (Lys-dendrimer) for comparison. This dendrimer was studied in our earlier work^37,43^.

## Experimental

Lysine-based dendrimers of 2nd generation were synthesized by standard solid phase peptide synthesis (SPPS) (see Supplementary Information). Dendrimers were purified, analyzed and characterized as described previously^20^.

Some structural parameters of the dendrimers are presented in Table 1.

**Table 1:** Some parameters of dendrimers.

Dendrimer	G	*M*_*d*_, a.u.m.	*N*_*ter*_	*N*_*in*_	*N*_*ch*_	D*10^10^, m^2^/s	*R*_*h*_, nm	*ρ*, g/cm^3^
Lys-2Lys	2	5636	16	14	44	1.02	1.94	0.31
Lys-2Gly	2	3620	16	14	16	1.36	1.45	0.47
Lys	2	2028	16	14	16	1.49	1.33	0.34

*G* is the number of generation; *M*_*d*_ is the molecular weight of a dendrimer. *N*_*ter*_ is the number of terminal lysine segments containing two protonated NH_3_^+^ groups, *N*_*in*_ is the number of inner segments, and *N*_*ch*_ is the number of charged groups; *D* is the diffusion coefficient (measured at room temperature); *R*_*h*_ is the hydrodynamic radius and *ρ* is the effective density.

We dissolved samples in 0.17 M NaCl D_2_O. Concentrations of dendrimers in the solvent were 2.69 g/dl, 1.49 g/dl, and 1.46 g/dl for Lys, Lys-2Lys, and Lys-2Gly, respectively. All systems correspond to the condition of the dilute solution.

^1^H NMR measurements were performed on a Bruker Avance III 500 MHz spectrometer using a Bruker Diff 30 diffusion probe with a Great 1/60 A amplifier. The ^1^H spin-lattice relaxation times, *T*_1H,_ were acquired with an “inversion-recovery” sequence modified by the destructive gradient pulses at the beginning of the sequence (“spoiler recovery” sequence)^59^. Parameters for the pulses were 12–18 μs duration of π/2 pulse, 16 tau delays and a 3 s recycle time between scans. The diffusion coefficients were measured by a stimulated echo sequence with bipolar gradients^60^ to compensate for the effects of convection. The gradient pulse length and the diffusion time were set to 1 ms and 20 ms, respectively. We used 16 gradients and 16 scans for each gradient.

**Figure 3:**
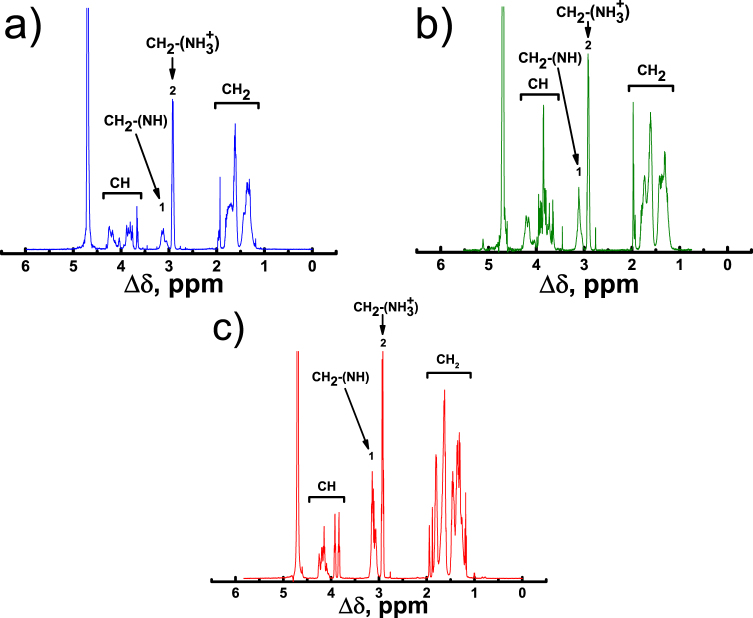
^1^Н NMR spectra of the Lys-2Lys (**a**), Lys-2Gly (**b**) and Lys (**c**) G2 dendrimers at T = 293 K. Peak 1 corresponds to inner СН_2_-(NH) and peak 2 to terminal CH_2_-(NH_3_^+^) groups.

We explored the signals from the СН_2_ groups chemically connected with N-atoms to study the orientational mobility in the samples by NMR relaxation because the signals from these groups are in the well-separated region of peptide dendrimer spectrum (chemical shift ~3 ppm), and because we can observe separate peaks for inner СН_2_-(NH) and terminal CH_2_-(NH_3_^+^) groups (peak 1 and peak 2 in Fig. 3, respectively)^37,43,58,61,62^. In the case of the Lys-2Lys dendrimer (Fig. 3a), the terminal CH_2_-(NH_3_^+^) groups in the side fragments (Fig. 2c,d) move freely like those in terminal segments (Fig. 2e) and, consequently, have the same chemical shift.

We also checked that a possible hydrated shell of charged *NH*_3_ groups does not influence on *T*_1H_ for neighboring *CH*_2_ groups (see Sections 2 and 3 in Supplementary information).

## Results and Discussion

To study local orientational mobility in the peptide dendrimers we consider temperature dependences of ^1^H spin-lattice NMR relaxation time, *T*_1H_. In the framework of the dipole-dipole relaxation mechanism of ^1^H nuclei (protons) 1/*T*_1H_ function can be written as^63–67^:

1 $$1/{T}_{1H}={A}_{0}(J({\omega }_{H},{\tau }_{i})+4J(2{\omega }_{H},{\tau }_{i})),$$

where *ω*_*H*_ is the cyclic resonance frequency (2π*f*_0_) for ^1^H nuclei; *A*_0_ is a constant that does not depend on temperature and frequency; and *J* is the spectral density which corresponds to Fourier transform from *P*_2_ orientational autocorrelation functions averaged over groups contributing to a corresponding peak. In the general case, the spectral density function for ^1^H nuclei has the form:

2 $$J(n{\omega }_{H},{\tau }_{i})=\sum _{i}\frac{{C}_{i}{\tau }_{i}}{1+{({\tau }_{i}n{\omega }_{H})}^{2}},$$

where *τ*_*i*_ and *C*_*i*_ are *i*th correlation times and their contribution to *J*, respectively, and *n* = 1, 2. The correlation time is determined by Arrhenius dependence

3 $$\tau ={\tau }_{0}\exp (\frac{{E}_{a}}{{k}_{b}{\rm{T}}}),$$

where *E*_*a*_ is the activation energy for the chosen group, T and *k*_*b*_ are temperature and Boltzmann constant, respectively.

**Figure 4:**
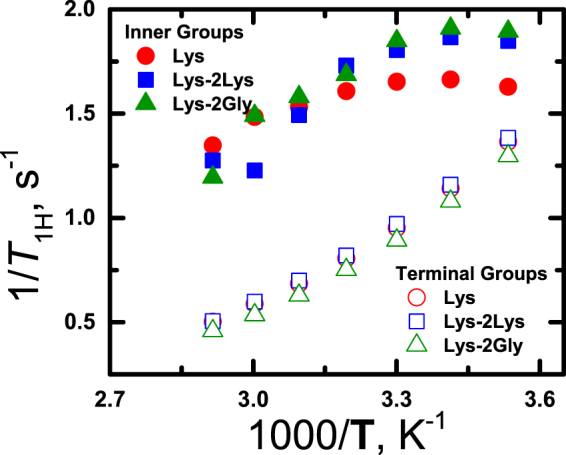
Temperature dependence of the spin-lattice relaxation rate, 1/T_1H_, of inner СН_2_-(NH) groups and terminal CH_2_-(NH_3_^+^) groups.

Figure 4 illustrates the experimental results for the temperature dependence of spin-lattice relaxation rate, 1/*T*_1H_. There is a significant difference between the 1/T_1H_ temperature dependences for peaks 1 (inner CH_2_-(NH) groups) and 2 (terminal CH_2_-(NH_3_^+^) groups) in the Lys-2Lys and Lys-2Gly, as well as for dendrimers with the simple repeating unit (Lys-dendrimer). The dispersion region (i.e., 1/T_1H_ maximum) is observed for the inner groups (peak 1), whereas for the terminal groups the 1/T_1H_ values increase exponentially with decreasing temperature. Thus, the terminal groups have higher mobility than inner segments^37^.

The temperature dependences of 1/*T*_1H_ for inner groups in the Lys-2Lys and Lys-2Gly dendrimers practically coincide. Therefore, the local mobility of the inner groups in the Lys-2Lys and Lys-2Gly is practically the same. This fact confirms the computer simulations results^38^ that NMR relaxation in dendrimer is not sensitive to EV effects because the density in these dendrimers is significantly different. To demonstrate the density difference in Lys-2Lys and Lys-2Gly dendrimers we measured their diffusion coefficients and calculated their hydrodynamic radii, *R*_*h*_, using the Stokes-Einstein equation (3)

4 $$D=\frac{{k}_{B}{\rm{T}}}{6\pi \eta {R}_{h}}$$

where *η* is the viscosity. If almost all atoms of the dendrimer concentrate in the sphere with a radius *R*_*h*_, we can estimate the density within the dendrimers, *ρ*, by using equation (4)

5 $$\rho =\frac{{M}_{d}}{\frac{4}{3}\pi {R}_{h}^{3}}$$

where *M*_*d*_ is the molecular weight of the dendrimer. The results of the calculation given in Table 1 shows that the density in Lys-2Gly is 1.5 times higher than in the Lys-2Lys.

We found it interesting that the maximum of 1/T_1H_ for inner groups in Lys dendrimer shifts to higher temperatures than the maxima of Lys-2Lys and Lys-2Gly dendrimers. This points out that the local mobility of the Lys-dendrimer inner segments is lower. The density of Lys-dendrimer is effectively the same as for Lys-2Lys. It means that the EV effects for both dendrimers are practically the same too; however, their relaxation rates are different. This result confirms the fact that volume effects are not the main and important factor in the NMR relaxation of inner dendrimer segments. According to the computer simulations^38^ based on the theory^34^, the main factor which effects on NMR relaxation is semiflexibility. The contour length of the Lys inner segment is shorter than that of the Lys-2Lys approximately in 3 times, and the decrease in the segment length leads to an increase in the semiflexibility. At the same time, the increase in the semiflexibility coefficient leads to the suppression of small-scale motions and to the increase of the contribution of the rotation of the dendrimer as a whole^37,38^. Thus, the increase of rigidity will result in a shift of the maximum to the low frequencies area (or high temperatures), that we observe for Lys in Fig. 4.

As mentioned above, the temperature dependence of 1/*T*_1H_ for peak 2 (terminal CH_2_-(NH_3_^+^) groups) in the dendrimers under study increases exponentially with temperature decrease and we can observe narrow region where *ωτ* << 1 for each correlation time. In this region, 1/*T*_1H_ can be proportional to

6 $$\frac{1}{{T}_{1H}}\approx {\tau }_{\max }$$

where *τ*_*max*_ is the maximal relaxation time of macromolecules. According to Eq. (6), a larger value of 1/*T*_1H_ corresponds to a slower local orientation mobility of the NMR active group.

As seen in Fig. 4, the 1/*T*_1H_ values for peak 2 for all three dendrimers differ only slightly. This can be the fact that the peak 2 is determined by the contribution of terminal groups, and the mobility of terminal groups is practically independent of size and macromolecule tree-like structure, except the Lys-2Lys dendrimer. In the case of the Lys-2Lys dendrimer, peak 2 is the sum of the contribution of terminal groups (33.3%) and side fragments (66.6%). The small difference of 1/*T*_1H_ dependence for Lys-2Lys from analogous 1/*T*_1H_ dependences for other dendrimers means that the orientational mobility of the groups in the side fragments coincides with the mobility of the terminal groups. Otherwise, a more complex temperature behavior (superposition of the peaks 1 and 2) of 1/*T*_1H_ should be observed for the Lys-2Lys dendrimer. This conclusion agrees well with results of the analytic theory^55^.

As a summary, our experimental results show that NMR relaxation of groups in semiflexible dendrimers is not sensitive to changes in volume interactions and is determined by a topological distance from the periphery. We believe that this confirmation of theoretical conclusions is important for the development of the theory of the orientational mobility of hyperbranched systems. Additionally, our results will be useful for biomedical applications of peptide dendrimers.

## Electronic supplementary material

Supplementary Information

## References

[1] Martinho N (2017). Rational design of novel, fluorescent, tagged glutamic acid dendrimers with different terminal groups and in silico analysis of their properties. International Journal of Nanomedicine.

[2] Hsu HJ, Bugno J, Lee SR, Hong S (2017). Dendrimer-based nanocarriers: a versatile platform for drug delivery. Wiley Interdisciplinary Reviews: Nanomedicine and Nanobiotechnology.

[3] Palmerston M. L., Pan, J. & Torchilin, V. Dendrimers as Nanocarriers for Nucleic Acid and Drug Delivery in Cancer Therapy. *Molecules* **22**, 1401 (2017). 10.3390/molecules22091401 PMC5600151 28832535

[4] Kuanga T (2016). Recent Progress in Dendrimer-based Gene Delivery Systems. Current Organic Chemistry.

[5] Singh, S. K. & Sharma, V. K. Dendrimers: A Class of Polymer in the Nanotechnology for Drug Delivery. *Nanomedicine for Drug Delivery and Therapeutics* 373–409, 10.1002/9781118636299.ch13 (2009).

[6] Sadler K, Tam (2002). J. P. Peptide dendrimers: applications and synthesis. Reviews in Molecular Biotechnology.

[7] Aharoni SM, Crosby CR, Walsh EK (1982). Size and Solution Properties of Globular tert-Butyloxycarbonyl-poly(α,ϵ-l-lysine). Macromolecules.

[8] Murthy NS, Aharoni SM (1983). Experimental Observation of the Onset of Finite Domain Boundaries in a Simple Two-Phase System by Small-Angle X-ray Scattering. Macromolecules.

[9] Roberts BP, Krippner GY, Scanlon MJ, Chalmers DK (2009). Molecular dynamics of variegated polyamide dendrimers. Macromolecules.

[10] Falkovich S, Markelov D, Neelov I, Darinskii A (2013). Are structural properties of dendrimers sensitive to the symmetry of branching? Computer simulation of lysine dendrimers. Journal of Chemical Physics.

[11] Neelov IM (2013). Molecular Properties of Lysine Dendrimers and their Interactions with A&beta;-Peptides and Neuronal Cells. Current Medicinal Chemistry.

[12] Rahimi A, Amjad-Iranagh S, Modarress H (2016). Molecular dynamics simulation of coarse-grained poly(L-lysine) dendrimers. Journal of Molecular Modeling.

[13] Mikhailov IV, Darinskii AA (2014). Does symmetry of branching affect the properties of dendrimers?. Polymer Science Series A.

[14] Neelov I, Adolf B (2003). D. Brownian Dynamics Simulation of Dendrimers under Elongational Flow: Bead-Rod Model with Hydrodynamic Interactions. Macromolecules.

[15] Ennari J, Elomaa M, Neelov I, Sundholm F (2000). Modeling of water-free and water containing solid polyelectrolytes. Polymer.

[16] Ennari J, Neelov I, Sundholm F (2000). Molecular dynamics simulation of the PEO sulfonic acid anion in water. Computational and Theoretical Polymer Science.

[17] Neelov IM, Binder K (1995). Brownian dynamics of grafted polymer chains: time dependent properties. Macromolecular Theory and Simulations.

[18] Tam JP (1988). Synthetic peptide vaccine design: Synthesis and properties of a high-density multiple antigenic peptide system. Proceedings of the National Academy of Sciences.

[19] Rao C, Tam JP (1994). Synthesis of Peptide Dendrimer. Journal of the American Chemical Society.

[20] Vlasov GP (2004). Lysine Dendrimers and Their Starburst Polymer Derivatives: Possible Application for DNA Compaction and *in vitro* Delivery of Genetic Constructs. Russian Journal of Bioorganic Chemistry.

[21] Gu ZW, Luo K, She WC, Wu Y, He B (2010). New-generation biomedical materials: Peptide dendrimers and their application in biomedicine. Science China Chemistry.

[22] Delort E, Darbre T, Reymond J-L (2004). A strong positive dendritic effect in a peptide dendrimer-catalyzed ester hydrolysis reaction. Journal of the American Chemical Society.

[23] Javor S, Delort E, Darbre T, Reymond JL (2007). A peptide dendrimer enzyme model with a single catalytic site at the core. Journal of the American Chemical Society.

[24] Polcyn P (2013). Novel antimicrobial peptide dendrimers with amphiphilic surface and their interactions with phospholipids - Insights from mass spectrometry. Molecules.

[25] Pires J (2015). *In Vitro* Activity of the Novel Antimicrobial Peptide Dendrimer G3KL against Multidrug-Resistant Acinetobacter baumannii and Pseudomonas aeruginosa. Antimicrobial Agents and Chemotherapy.

[26] Manikkath J, Hegde AR, Kalthur G, Parekh HS, Mutalik S (2017). Influence of peptide dendrimers and sonophoresis on the transdermal delivery of ketoprofen. International Journal of Pharmaceutics.

[27] Kwok A, Eggimann GA, Reymond JL, Darbre T, Hollfelder F (2013). Peptide dendrimer/lipid hybrid systems are efficient DNA transfection reagents: Structure-activity relationships highlight the role of charge distribution across dendrimer generations. ACS Nano.

[28] Kwok A (2016). Efficient Transfection of siRNA by Peptide Dendrimer–Lipid Conjugates. ChemBioChem.

[29] Santos SS (2017). Peptide dendrimers: drug/gene delivery and other approaches. Canadian Journal of Chemistry.

[30] Javor S, Reymond J (2009). Molecular Dynamics and Docking Studies of Single Site Esterase Peptide Dendrimers Supporting information. J. Org. Chem..

[31] Filipe LCS, Machuqueiro M, Darbre T, Baptista AM (2016). Exploring the Structural Properties of Positively Charged Peptide Dendrimers. The Journal of Physical Chemistry B.

[32] Filipe LCS, Campos SRR, Machuqueiro M, Darbre T, Baptista AM (2016). Structuring peptide dendrimers through pH modulation and substrate binding. Journal of Physical Chemistry B.

[33] Kimmich, R. & Fatkullin, N. In *Advances in Polymer Science* (eds. Fatkullin, N. *et al*.) 1–113 (Springer Berlin Heidelberg, 2004). 10.1007/978-3-540-40000-4_1.

[34] Markelov DA, Dolgushev M, Gotlib YY, Blumen A (2014). NMR relaxation of the orientation of single segments in semiflexible dendrimers. Journal of Chemical Physics.

[35] Karatasos K, Adolf DB, Davies GR (2001). Statics and dynamics of model dendrimers as studied by molecular dynamics simulations. The Journal of Chemical Physics.

[36] Markelov DA (2009). Orientational mobility and relaxation spectra of dendrimers: Theory and computer simulation. J. Chem. Phys..

[37] Markelov DA (2015). Molecular dynamics simulation of spin–lattice NMR relaxation in poly-L-lysine dendrimers: manifestation of the semiflexibility effect. Phys. Chem. Chem. Phys..

[38] Shavykin, O. V., Neelov, I. M. & Darinskii, A. A. Is the manifestation of the local dynamics in the spin-lattice NMR relaxation in dendrimers sensitive to excluded volume interactions? *Phys. Chem. Chem. Phys.* **18**, 24307–24317 (2016). 10.1039/c6cp01520d 27531617

[39] Novoa-Carballal R (2010). The dynamics of GATG glycodendrimers by NMR diffusion and quantitative 13C relaxation. Phys. Chem. Chem. Phys..

[40] Markelov DA (2010). NMR studies of carbosilane dendrimer with terminal mesogenic groups. Journal of Physical Chemistry B.

[41] Pinto LF, Correa J, Martin-Pastor M, Riguera R, Fernandez-Megia E (2013). The dynamics of dendrimers by NMR relaxation: Interpretation pitfalls. Journal of the American Chemical Society.

[42] Pinto LF, Riguera R, Fernandez-Megia E (2013). Stepwise filtering of the internal layers of dendrimers by transverse-relaxation-edited NMR. Journal of the American Chemical Society.

[43] Neelov IM (2013). Mathematical simulation of lysine dendrimers: Temperature dependences. Polymer Science Series C.

[44] Markelov, D. A., Dolgushev, M. & Lähderanta, E. In *Annual Reports on NMR Spectroscopy* (ed. Webb, G. A.) **91**, 1–66 (Academic Press, 2017).

[45] Dolgushev M, Schnell S, Markelov DA (2017). Local NMR Relaxation of Dendrimers in the Presence of Hydrodynamic Interactions. Applied Magnetic Resonance.

[46] Hofmann M (2015). Field-Cycling Relaxometry as a Molecular Rheology Technique: Common Analysis of NMR, Shear Modulus and Dielectric Loss Data of Polymers vs Dendrimers. Macromolecules.

[47] Mohamed F, Hofmann M, Pötzschner B, Fatkullin N, Rössler EA (2015). Dynamics of PPI Dendrimers: A Study by Dielectric and H-2 NMR Spectroscopy and by Field-Cycling H-1 NMR Relaxometry. Macromolecules.

[48] Markelov DA (2016). Orientational Mobility in Dendrimer Melts: Molecular Dynamics Simulations. Macromolecules.

[49] Matveev VV (2017). Investigation of Melts of Polybutylcarbosilane Dendrimers by 1H NMR Spectroscopy. Scientific Reports.

[50] Gao C, Yan D (2004). Hyperbranched polymers: from synthesis to applications. Progress in Polymer Science.

[51] Voit BI, Lederer A (2009). Hyperbranched and Highly Branched Polymer Architectures—Synthetic Strategies and Major Characterization Aspects. Chemical Reviews.

[52] Lederer, A. & Burchard, W. In *Hyperbranched Polymers: Macromolecules in Between Deterministic Linear Chains and Dendrimer Structures* 1–286 (Royal Soc. Chemistry, 2015).

[53] Neelov IM, Adolf DB (2004). Brownian Dynamics Simulation of Hyperbranched Polymers under Elongational Flow. The Journal of Physical Chemistry B.

[54] Qi Y, Dolgushev M, Zhang Z (2014). Dynamics of semiflexible recursive small-world polymer networks. Scientific Reports.

[55] Grimm J, Dolgushev M (2016). Dynamics of internally functionalized dendrimers. Physical Chemistry Chemical Physics.

[56] Dolgushev M, Markelov DA, Fürstenberg F, Guérin T (2016). Local orientational mobility in regular hyperbranched polymers. Physical Review E.

[57] Jurjiu, A., Biter, T.-L. & Turcu, F. Dynamics of a Polymer Network Based on Dual Sierpinski Gasket and Dendrimer: A Theoretical Approach. *Polymers* **9** (2017). 10.3390/polym9070245 PMC6432022 30970922

[58] Harada, A., Matsuki, R., Ichimura, S., Yuba, E. & Kono, K. Intracellular Environment-Responsive Stabilization of Polymer Vesicles Formed from Head-Tail Type Polycations Composed of a Polyamidoamine Dendron and Poly(L-lysine). *Molecules* **18** (2013). 10.3390/molecules181012168 PMC6269863 24084020

[59] Sorland, G. H. In *Dynamic Pulsed-Field-Gradient NMR* **110**, 169–190 (Springer-Verlag Berlin, 2014).

[60] Jerschow A, Müller N (1996). 3D Diffusion-Ordered TOCSY for Slowly Diffusing Molecules. Journal of Magnetic Resonance, Series A.

[61] Zhang Y-X, Chen Y-F, Shen X-Y, Hu J-J, Jan J-S (2016). Reduction- and pH-Sensitive lipoic acid-modified Poly(l-lysine) and polypeptide/silica hybrid hydrogels/nanogels. Polymer.

[62] Piedra-Arroni, E. *et al*. Smart Poly(imidazoyl-l-lysine): Synthesis and Reversible Helix-to-Coil Transition at Neutral pH. *Polymers* **9** (2017). 10.3390/polym9070276 PMC6432093 30970954

[63] Abragam, A. *The principles of nuclear magnetism*. (Clarendon Press; Oxford University Press: Oxford [Oxfordshire], 1983).

[64] Bloembergen N, Purcell EM, Pound RV (1948). Relaxation effects in nuclear magnetic resonance absorption. Physical Review.

[65] Solomon I (1955). Relaxation processes in a system of two spins. Physical Review.

[66] Chizhik, V. I. *et al*. *Magnetic Resonance and Its Applications*. (Springer, 2014). 10.1007/978-3-319-05299-1.

[67] Levitt, M. H. *Spin dynamics: basics of NMR*, *2nd edn*. (Wiley, New York, 2008).

