# Supplementary material for: NMR studies of excluded volume interactions in peptide dendrimers
Source: Sci Rep. 2018 Jun 11;8:8916. doi: 10.1038/s41598-018-27063-3 (PMC5995971; doi:10.1038/s41598-018-27063-3)
Supplement: Supplementary file 1 — Supplementary Information [file 41598_2018_27063_MOESM1_ESM.pdf]

Supplementary Information

NMR studies of excluded volume interactions in peptide dendrimers

Nadezhda N. Sheveleva,[a] *Denis A. Markelov,*[a*] *Mikhail A. Vovk,*[a] *Maria E. Mikhailova,*[a] *Irina I. Tarasenko,*[b] *Igor M. Neelov,*[c] *Erkki Lähderanta*[d]

[a]*St. Petersburg State University, 7/9 Universitetskaya nab., St. Petersburg, 199034 Russia,*

[b]*Institute of Macromolecular Compounds, Russian Academy of Sciences, Bolshoi Prospect 31, V.O., St. Petersburg, 199004 Russia.*

[c]*St. Petersburg National Research University of Information Technologies, Mechanics and Optics (ITMO University), Kronverkskiy pr. 49, St. Petersburg, 197101 Russia.*

[d]*Laboratory of Physics, Lappeenranta University of Technology, Box 20, 53851 Lappeenranta, Finland*

1. Synthesis of Lys-2Lys and Lys-2Gly dendrimers

Materials. Amino acids (L-lysine, L-glycine, L-alanine) were obtained from "Iris Biotech GMBH" (Germany); trifluoromethanesulfonicacid (TFMSA), diisipropylcarbodiimide (DIC), 1-hydroxybenzotriazole (HOBt), thioanisole, ethanedithiol and other reagents were purchased from Sigma–Aldrich (Germany) and used as received. Triethylamine, dichloromethane were purchased from Vecton Ltd. (Russia) and distilled prior to use. Dimethylformamide (DMF), also purchased from Vecton Ltd. (Russia), was

[*] Corresponding Author, Email: markeloved@gmail.com

dried under molecular sieves 4 Å and distilled under vacuum. Trifluoroacetic acid (TFA) purchased from Panreac (Spain) was distilled before application. All solvents were purified and distilled using standard procedures.

Instruments. Reaction products were analyzed by reversed-phase HPLC on a chromatograph Shimadzu LC-20 Prominence system (Japan) and the Luna C18(2), 4.6 × 150 mm column, 5μ mm, for analytical chromatography and Discovery C18 21.6 × 250 mm, 5μ for the preparative.

Synthesis and characterization. Lysine-based dendrimers were synthesized by standard solid phase peptide synthesis (SPPS), performed on a polymer support, p-methylbenzhydrylamine resin (Bachem Biochemica GmbH) using the BOC-strategy, DIC/HOBt as a condensing mixture, and trifluoroacetic acid for deblocking at the acylation stage. To protect functional groups of amino acids, the tert-butylhydroxycarbonyl (Boc), and 2- chlorobensylhydroxycarbonyl groups were used. At the C-terminus of the dendrimers of this series, an alanine (Ala) residue was introduced so that the amino acid analysis of the synthesized dendrimers could reasonably monitor their structure. Nε,Nα-di-(tert-butylhydroxycarbonyl)lysine was introduced into the branching point and, subsequently, doubled amounts of derivative amino acids were added. The excess of the reagent introduced into the reaction was generally 3-4 equivalents per amino group based on the initial capacity of the polymeric carrier. The completeness of the acylation reaction was controlled by the presence of free amino groups using the Kaiser test (ninhydrin test). The protective BOC- group was removed during the synthesis by the action of trifluoroacetic acid (TFA). The final stage of the synthesis was the cleavage of the target dendrimer molecule from the polymeric carrier with the simultaneous complete

deprotection of the trifluoromethanesulfonic acid/trifluoroacetic acid (TMSA/TFA) system in the presence of scavengers. Isolation and purification of the target molecules was carried out by chromatographic methods.

The following main stage was used to obtain 0.2 g of p-methylbenzhydrylamine resin (capacity 0.85 mmol/g): (1) deprotection, 50% TFA/CH2Cl2 (5ml), 20 min; (2) deprotonation, 10% Et3N/DMF (5ml x 2), 15 min; (3) coupling, 1.5 mM Boc-aminoacid, 15 mM DIC, 1.5 mM HOBt/DMF (5ml), 2 h; and a ninhydrin test. For this, the resin was washed with dimethylformamide and dichloromethane. In the case of the incomplete coupling (positive ninhydrin reaction) the protocol was repeated from stage (2).

Note that the problem of exhaustive acylation of the branched oligolysyl polymer appeared when passing from a dispherical to a trispherical construct in the process of dendrimers Lys-2Gly and Lys-2Lys synthesis: two acylations with the activated derivative of N,N-di-(tert-butyloxycarbonyl)lysine and an increase in the acylation time were necessary to complete the reaction. And reaction times were prolonged for the amino acids after one branching unit (180 min), after two branching units (240 min), and three branching units (360 min)); the equivalents of amino acid, DIC, HOBt were accordingly increased. The last stage of the growth of the dendrimer was necessary to add 4-N,N-dimethylaminopyridine (DMAP) to the reaction mixture as a catalyst for complete conversion.

The complete deprotection and splitting off of the dendrimer from polymer was carried out using trifluoromethanesulfonic acid (1 ml) in TFA (10 ml) in the presence of thioanisole (1 ml) and ethanedithiol (0.5 ml) for 1 h at 0°C and then 1.5 h at room temperature. The mixture was diluted with ethyl ether (30 ml) and filtered. The dendrimer

was dissolved in TFA (10 ml), filtered to remove the resin, and precipitated by dry ether (100 ml).

The crude dendrimers Lys-2Gly and Lys-2Lys were isolated by gel-filtration on Sephadex G-50 column (2.5 x 50 cm) using 10% acetic acid as eluent and purified by RP-HPLC in the system “water–acetonitrile–0.1% trifluoroacetic acid” with the use of linear ascending acetonitrile gradient. The isolated fractions had 95% purity by counting from the basic line (the UV detection was performed at 230 nm). After lyophilization of the corresponding fractions, the purification degree of product was analyzed by RP-HPLC.

2. Diffusion measurements

The diffusion coefficients of all the dendrimer groups (including inner CH_2-(NH) and terminal CH_2-(NH_3^+) groups) are very close.

Table S1. *The values of diffusion coefficients for the groups in Lys-2Lys, Lys-2Gly and Lys dendrimers. The numbering of peaks corresponds to Fig. S1.*

Peak number	Diffusion coefficient, D*10, m^2/s		
	Lys-2Lys	Lys-2Gly	Lys
1	0.95	1.37	1.58
2	1.00	1.29	1.46
3	1.12	1.51	1.51
4	0.95	1.32	1.48
5	1.13	1.40	1.45
6	1.06	1.34	1.52

7	0.96	1.34	1.47

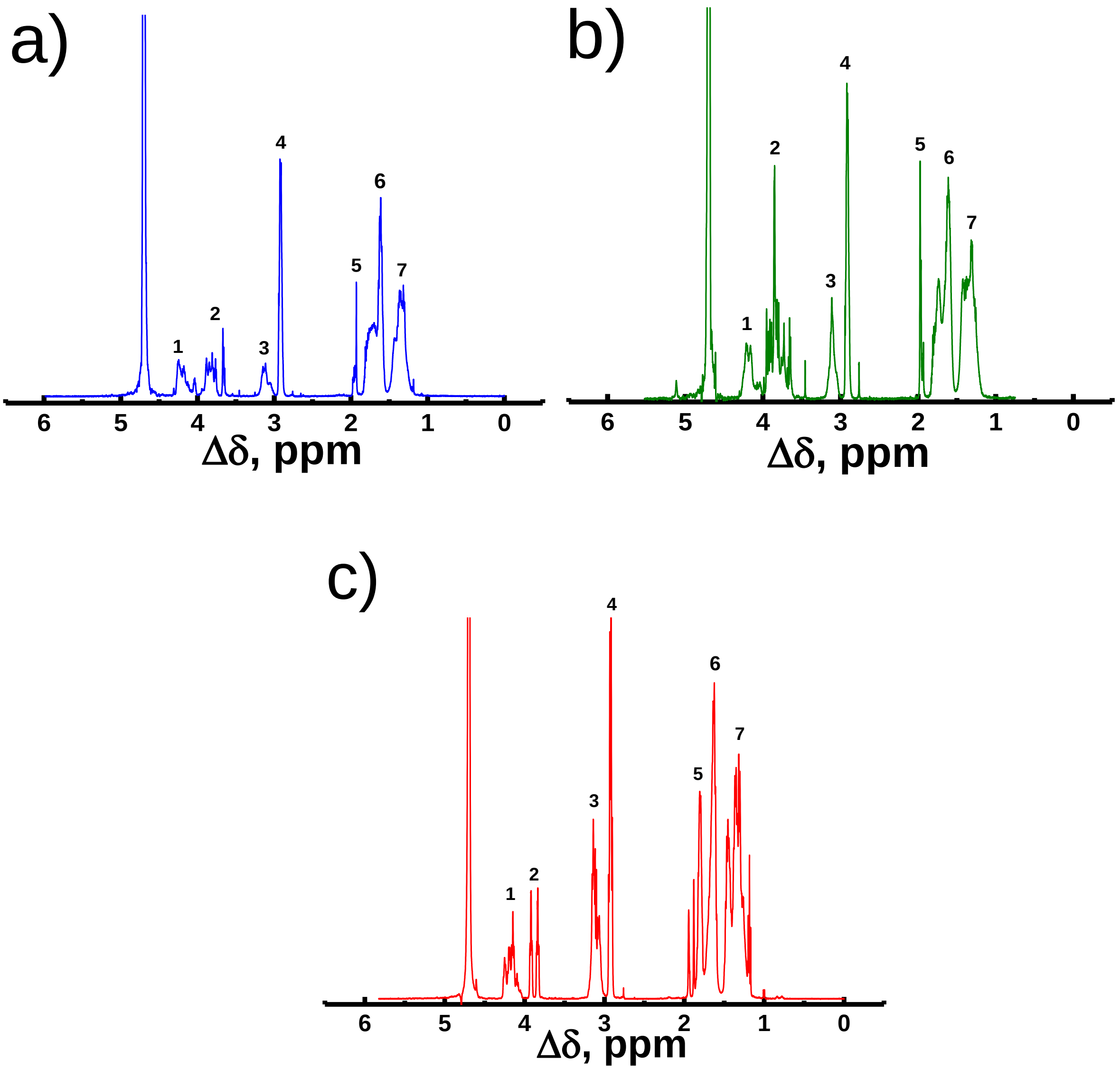

Figure S1. *^{1}H NMR spectra of Lys-2Lys (a), Lys-2Gly (b) and Lys (c) G2 dendrimers. The numbers on the spectra correspond to the peak numbers in the Table S1.*

3. NOESY measurements

There is no cross-peak between the water protons and CH_2-(NH_3^+) groups in the NOESY spectrum (see Fig. S2). Also we have performed the selective ^{1}H-^{1}H NOESY experiment. The 1D

selective NOESY spectrum (Fig. S3) shows that the water protons do not interact with the protons of the CH_2-(NH_3^+) groups in Lys-dendrimer.

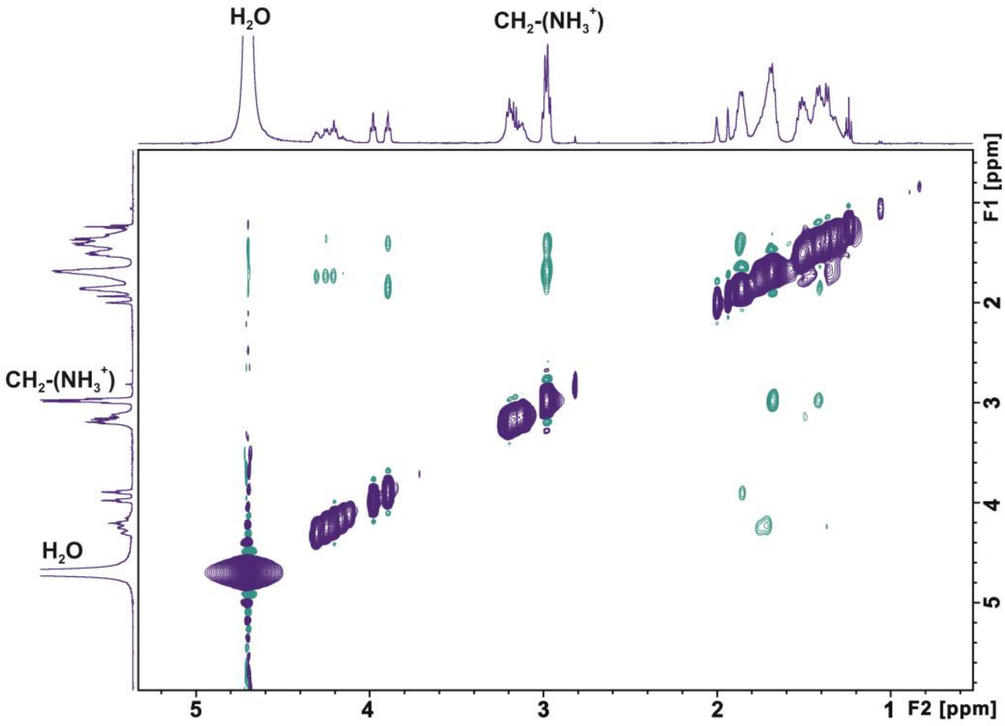

Figure S2. *^1H-^1H NOESY NMR spectrum of Lys-dendrimer.*

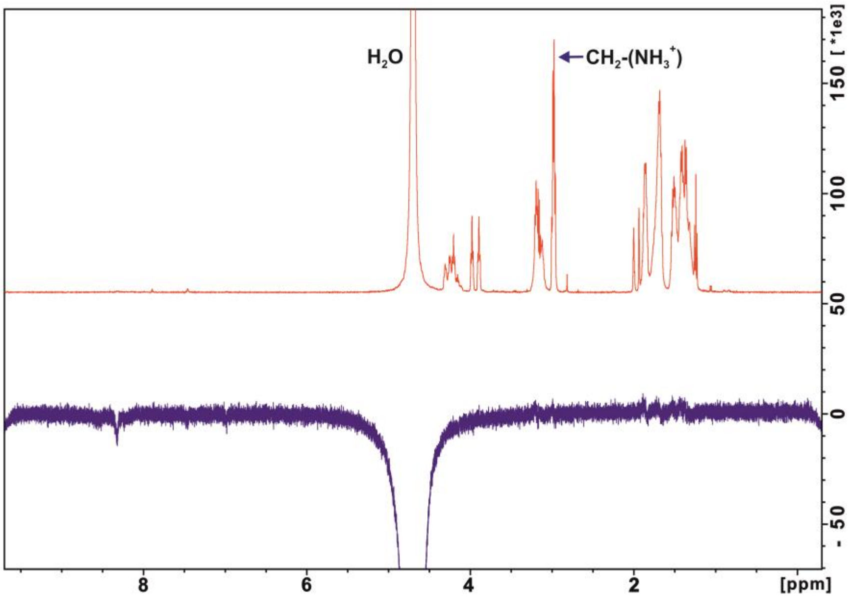

Figure S3. *1D selective NOESY spectrum of Lys dendrimer with excitation of water protons.*
